# Early oxygen levels contribute to brain injury in extremely preterm infants

**DOI:** 10.1038/s41390-021-01460-3

**Published:** 2021-03-22

**Authors:** Krista Rantakari, Olli-Pekka Rinta-Koski, Marjo Metsäranta, Jaakko Hollmén, Simo Särkkä, Petri Rahkonen, Aulikki Lano, Leena Lauronen, Päivi Nevalainen, Markus J. Leskinen, Sture Andersson

**Affiliations:** 1 Children’s Hospital, Pediatric Research Center, Helsinki University Hospital, University of Helsinki, Helsinki, Finland; 2 Department of Computer Science, Aalto University School of Science, Espoo, Finland; 3 Department of Computer and Systems Sciences, Stockholm University, Stockholm, Sweden; 4 Pediatric Neurology, Children’s Hospital, Helsinki University Hospital, University of Helsinki, Helsinki, Finland; 5 Clinical Neurophysiology, HUS Medical Imaging Center, Children’s Hospital, Helsinki University Hospital, University of Helsinki, Helsinki, Finland; 6 BioMag Laboratory, HUS Medical Imaging Center, Helsinki University Hospital, University of Helsinki, Helsinki, Finland

## Abstract

**Background:**

Extremely low gestational age newborns (ELGANs) are at risk of neurodevelopmental impairments that may originate in early NICU care. We hypothesized that early oxygen saturations (SpO_2_), arterial pO_2_ levels, and supplemental oxygen (FiO_2_) would associate with later neuroanatomic changes.

**Methods:**

SpO_2_, arterial blood gases, and FiO_2_ from 73 ELGANs (GA 26.4 ± 1.2; BW 867 ± 179 g) during the first 3 postnatal days were correlated with later white matter injury (WM, MRI, *n* = 69), secondary cortical somatosensory processing in magnetoencephalography (MEG-SII, *n* = 39), Hempel neurological examination (*n* = 66), and developmental quotients of Griffiths Mental Developmental Scales (GMDS, *n* = 58).

**Results:**

The ELGANs with later WM abnormalities exhibited lower SpO_2_ and pO_2_ levels, and higher FiO_2_ need during the first 3 days than those with normal WM. They also had higher pCO_2_ values. The infants with abnormal MEG-SII showed opposite findings, i.e., displayed higher SpO_2_ and pO_2_ levels and lower FiO_2_ need, than those with better outcomes. Severe WM changes and abnormal MEG-SII were correlated with adverse neurodevelopment.

**Conclusions:**

Low oxygen levels and high FiO_2_ need during the NICU care associate with WM abnormalities, whereas higher oxygen levels correlate with abnormal MEG-SII. The results may indicate certain brain structures being more vulnerable to hypoxia and others to hyperoxia, thus emphasizing the role of strict saturation targets.

**Impact:**

- This study indicates that both abnormally low and high oxygen levels during early NICU care are harmful for later neurodevelopmental outcomes in preterm neonates.
- Specific brain structures seem to be vulnerable to low and others to high oxygen levels.
- The findings may have clinical implications as oxygen is one of the most common therapies given in NICUs.
- The results emphasize the role of strict saturation targets during the early postnatal period in preterm infants.

## Introduction

Extremely low gestational age newborns (ELGANs), born before 28 weeks of gestational age (GA), are at risk of brain injury and later neurodevelopmental complications.^1–8^ Many of these complications are thought to originate during their early care, when they are exposed to various factors, such as oxygen, which is one of the most common therapies given in neonatal intensive care units (NICUs).^1,2,7,9–14^ Inappropriately low oxygen levels are, e.g., associated with increased mortality and impaired neurodevelopment,^9,15,16^ and higher levels with retinopathy and lung injury.^16^ However, despite intensive research, the long-term effects of early oxygen levels in preterm infants are incompletely characterized.^7,10–12^

Medical data from electronic health record systems and monitoring devices are increasingly available and methods for analyzing large data sets have improved, making analysis of NICU information system data feasible. As a part of our multimethodological study of ELGANs, we correlated oxygen-related parameters from the first 3 postnatal days of life with neuroimaging (magnetic resonance imaging (MRI)) and magnetoencephalography (MEG) at term equivalent age (TEA) and neurodevelopmental outcomes at 2 years of corrected age. The main outcomes were white matter (WM) injury in brain MRI and changes in secondary cortical somatosensory processing measured by MEG-SII, as these abnormalities may originate during the neonatal phase.^8,17–23^ Neurodevelopmental outcome was assessed by Hempel neurological examination and the Griffiths Mental Developmental Scales (GMDS).

We hypothesized that, in ELGANs aberrant oxygen saturations (SpO_2_), lower arterial partial pressures of oxygen (pO_2_), and higher oxygen demand (as measured by fraction of inspired oxygen (FiO_2_)) during the first 3 postnatal days would correlate with pathologic changes in neuroanatomical structures and unfavorable neurodevelopmental outcomes.

## Patients and methods

### Patients

**Fig. 1:**
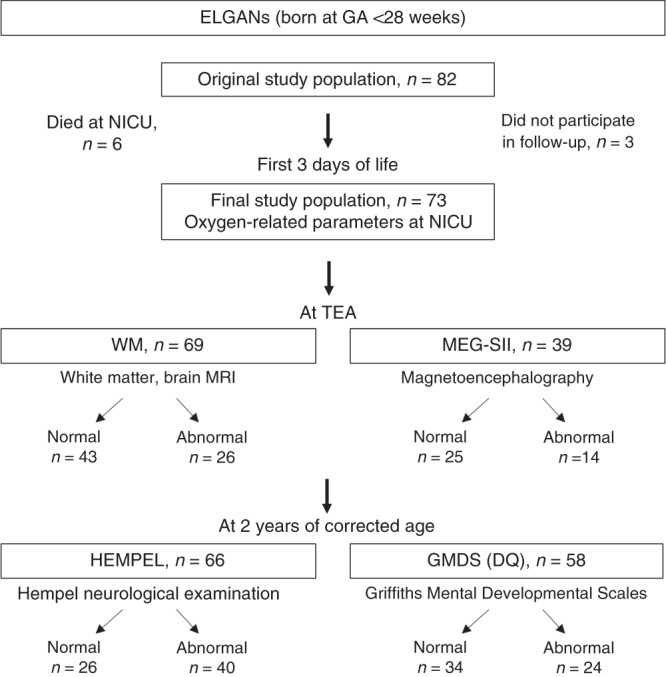
Study design. DQ developmental quotient, ELGAN extremely low gestational age newborn, preterm babies born before 28 weeks of GA, GA gestational age, GMDS Griffiths Mental Developmental Scales, MEG-SII secondary cortical somatosensory processing in magnetoencephalography, HEMPEL Hempel neurological examination, NICU neonatal intensive care unit, TEA term equivalent age, WM white matter, white matter injury in MRI.

The original patient group consisted of 82 ELGANs, who were born before 28 gestational weeks and treated in level IV NICU at Children’s Hospital, Helsinki, Finland. They were recruited for a multimethodological study between 5/2006 and 9/2008 and the parents provided signed informed consents. All clinical decisions were made by existing treatment protocols. The SpO_2_ target range was 90–95%, the blood transfusion range was hematocrit (HCT) <40% when on ventilator care and/or needing supplemental oxygen, and HCT <30% for more stable infants. No delayed cord clamping was performed at birth. Six infants died during the NICU period and three infants did not participate in the follow-up examinations. Consequently, the final study population included 73 ELGANs (Fig. 1). The clinical characteristics of the infants are presented in Table 1A, B. The ethics committee of the Hospital District of Helsinki and Uusimaa, Finland approved the study protocols (Dnro HUS 277/E7/2005, 2008 and HUS 115/13/03/00/2014).

**Table 1:** (A) Clinical characteristics of ELGANS with normal and abnormal white matter (WM) and secondary cortical somatosensory processing in magnetoencephalography (MEG-SII) at term equivalent age. (B) Clinical characteristics of ELGANs with normal and abnormal Hempel examination and developmental quotients (DQ) of Griffiths Mental Developmental Scales (GMDS) at 2 years of corrected age.

(A)							
Patients	All (*n* = 73)	WM (*n* = 69)			MEG-SII (*n* = 39)		
		Norm.	Abnorm.	*p* value	Norm.	Abnorm.	*p* value
*n*/total (*n*, %)	73	43/69 (62%)	26/69 (38%)		25/39 (64%)	14/39 (36%)	
GA at birth (weeks; mean, SD)	26.4 (1.2)	26.5 (1.1)	26.1 (1.3)	0.216	26.5 (1.3)	26.6 (1.0)	0.727
Birth weight (g; mean, SD)	867 (179)	866 (175)	868 (190)	0.955	855 (175)	936 (185)	0.182
SGA, <2 SD (*n*, %)	15 (21%)	10 (23%)	4 (15%)	0.213	8 (32%)	2 (14%)	0.163
Male (*n*, %)	46 (63%)	22 (51%)	21 (81%)	**0.014**	10 (40%)	10 (71%)	0.062
Twin (*n*, %)	17 (23%)	7 (16%)	9 (35%)	0.082	5 (20%)	4 (29%)	0.554
Umbilical artery pH (mean, SD)	7.29 (0.08)	7.29 (0.06)	7.27 (0.11)	0.442	7.30 (0.06)	7.31 (0.06)	0.657
1-min Apgar (score; median, CI)	5 (4.5–5.5)	5 (4.4–5.6)	3 (2.0-4.0)	**0.067**	5 (5.1–5.9)	6 (5.0–7.0)	0.54
5-min Apgar (score; median, CI)	7 (6.5–7.5)	7 (6.5–7.5)	6 (5.1–6.9)	**0.081**	6 (5.6–7.4)	7 (6.0–8.0)	0.381
10-min Apgar (score; median, CI)	7 (6.7–7.3)	7 (6.7–7.3)	5 (4.5–5.5)	**0.053**	7 (6.6–7.4)	8 (7.4–8.6)	0.159
Antenatal steroid(s) (*n*, %)	72 (99%)	43 (100%)	25 (96%)	0.289	25 (100%)	14 (100%)	—
Postanal steroid (*n*, %)	23 (32%)	12 (28%)	11 (42%)	0.175	6 (24%)	4 (29%)	0.481
Surfactant (*n*, %)	73 (100%)	43 (100%)	26 (100%)	—	25 (100%)	14 (100%)	—
RDS (*n*, %)	52 (71%)	28 (65%)	23 (88%)	**0.033**	18 (72%)	7 (50%)	0.178
BPD at 36+0 GW (*n*, %)	33 (45%)	17 (40%)	15 (58%)	0.073	9 (36%)	6 (43%)	0.683
IVH, any (*n*, %)	30 (41%)	11 (26%)	18 (69%)	**0.001**	9 (36%)	4 (29%)	0.647
IVH, grades III–IV (*n*, %)	13 (18%)	2 (5%)	11 (42%)	**0.001**	4 (16%)	2 (14%)	0.89
Sepsis, (*n*, %)	37 (51%)	19 (44%)	16 (62%)	0.426	8 (32%)	5 (36%)	0.718
NEC (*n*, %)	6 (8%)	2 (5%)	3 (12%)	0.584	2 (8%)	1 (7%)	0.71
ROP (*n*)	22 (30%)	11 (26%)	10 (38%)	0.179	6 (24%)	2 (14%)	0.484

(B)						
Patients	Hempel (*n* = 66)			GMDS (DQ) (*n* = 58)		
	Norm.	Abnorm.	*p* value	Norm.	Abnorm.	*p* value
*n*/total (*n*, %)	26/66 (39%)	40/66 (61%)		34/58 (59%)	24/58 (41%)	
GA at birth (weeks; mean, SD)	26.3 (1.2)	26.4 (1.2)	0.682	26.5 (1.1)	26.1 (1.2)	0.252
Birth weight (g; mean, SD)	869 (178)	863 (195)	0.897	869 (174)	819 (184)	0.3
SGA, <2 SD (*n*, %)	5 (19%)	9 (23%)	0.645	9 (26%)	5 (21%)	0.778
Male (*n*, %)	13 (46%)	30 (75%)	**0.017**	19 (56%)	16 (67%)	0.417
Twin (*n*, %)	9 (35%)	7 (18%)	0.116	9 (26%)	3 (13%)	0.202
Umbilical artery pH (mean, SD)	7.29 (0.06)	7.29 (0.09)	0.897	7.28 (0.06)	7.3 (0.11)	0.366
1-min Apgar (score; median, CI)	4 (3.2–4.8)	5.5 (4.8–6.2)	0.968	5 (4.3–5.7)	5.5 (4.6–6.4)	0.97
5-min Apgar (score; median, CI)	7 (6.2–7.8)	7 (6.4–7.6)	0.73	7 (6.3–7.7)	6 (5.1–6.9)	0.111
10-min Apgar (score; median, CI)	7 (6.6–7.4)	7 (6.6–7.4)	0.817	7 (6.7–7.3)	7 (6.5–7.5)	0.967
Antenatal steroid(s) (*n*, %)	26 (100%)	40 (100%)	0.485	34 (100%)	24 (100%)	0.982
Postanal steroid (*n*, %)	10 (38%)	10 (25%)	0.485	11 (32%)	7 (29%)	0.897
Surfactant (*n*, %)	26 (100%)	40 (100%)	—	34 (100%)	24 (100%)	—
RDS (*n*, %)	20 (77%)	27 (68%)	0.417	27 (79%)	14 (58%)	0.085
BPD at 36+0 GW (*n*, %)	15 (58%)	17 (43%)	0.234	16 (47%)	13 (54%)	0.601
IVH, any (*n*, %)	10 (38%)	15 (38%)	0.938	12 (35%)	7 (29%)	0.632
IVH, grades III–IV (*n*, %)	2 (8%)	9 (23%)	0.118	3 (9%)	4 (17%)	0.375
Sepsis (*n*, %)	13 (50%)	21 (53%)	0.41	16 (47%)	15 (63%)	0.234
NEC (*n*, %)	1 (4%)	5 (13%)	0.486	2 (6%)	2 (8%)	0.737
ROP (*n*)	5 (19%)	16 (40%)	0.079	7 (21%)	11 (46%)	**0.041**

Data are presented as *n* (%) or mean (SD) or median (95% CI).

Statistically significant *p*-values are in bold.

### NICU biosignal data and oxygen-related parameters

Data gathered during the NICU phase, including patient monitor data, data from ventilators and other medical devices, observation variables, laboratory results, and diagnoses were stored in Centricity Critical Care information system (GE Healthcare, Chicago, IL). Monitor and ventilator data including SpO_2_, FiO_2_, and heart rate (HR) were recorded automatically and stored as 2-min averages of median values for 10-s intervals. The observation variables include both background information (e.g., GA, mode of delivery, sex) and clinical measurements (e.g., SpO_2_, FiO_2_). Manual readings were entered into the database by staff and represent the value at the time of reading. The partial pressures of oxygen (pO_2_) and carbon dioxide (pCO_2_) in arterial blood were measured using routine blood gas analyzers.

### Brain MRI and determination of WM abnormalities

Brain MRI (1.5 T) including T2-weighted axial and T1-weighted 3D sagittal images was performed at TEA. Two experienced neuroradiologists classified the MRI images according to Woodward et al. with modifications.^17^ WM was classified based on five variables: WM signal abnormality, periventricular WM volume loss, cystic abnormalities, ventricular dilatation, and thinning of the corpus callosum. The thinning of corpus callosum had two grades (grade 1 was normal and grade 2 had thinning of the corpus callosum). All the other variables had three grades, grade 1 being normal and grade 3 having most severe abnormalities. Based on the total score, the WM MRI findings were classified as abnormal if the total score was ≥7. In a subgroup analysis, comparisons were performed between patients with normal WM and those with severe WM changes having total scores of ≥10.

### MEG and sensory evoked magnetic fields

MEG analysis was performed at TEA as previously described.^18^ MEG recording was performed using a whole-head adult-sized helmet-shaped sensor array consisting of 306 independent channels: 204 gradiometers and 102 magnetometers (Vector-view, Elekta Neuromag Oy, Helsinki, Finland). Electroencephalography and electro-oculography were recorded for sleep stage monitoring. The sensory evoked magnetic fields were elicited by tactile stimulus to the tip of the index finger by a thin elastic membrane expanded by an air pressure pulse delivered through a plastic tube (Somatosensory Stimulus Generator, 4-D NeuroImaging Inc., San Diego, CA) and the interstimulus interval was 2 s. The MEG data were analyzed as previously described.^18,19^ The SII response, peaking at about 200 ms after tactile stimulation, was defined as abnormal when it was absent after both contralateral and ipsilateral stimulation in at least one hemisphere (right or left).^18^

### Neurodevelopmental assessments

An experienced child neurologist performed the clinical neurodevelopmental assessments at 2 years of corrected age according to a structured Hempel neurological examination^24^ and GMDS.^25,26^ The Hempel neurological examination consisted of five functional domains: posture and muscle tone, gross motor function, fine motor function, reflexes, and visual system. For the present study, infants with any dysfunctional domain(s) were classified as abnormal and the patients with normal findings as normal. In a subgroup analysis, comparisons were performed between patients with normal Hempel assessments and those with major neurologic impairment (cerebral palsy (CP)).

The general developmental quotient (DQ) of GMDS was based on five subscales: locomotor, personal–social, hearing–language, eye–hand coordination, and performance. DQ and subscale quotients (SQs) were calculated on the basis of the raw scores and the corrected age of the child. DQ and SQ scores <−1 SD were defined as abnormal and the cut-off points for impairment were for general DQ 88.7, locomotor 84.3, personal–social 84.8, hearing–language 84.6, eye–hand coordination 84.3, and for performance 84.4. In addition to separate SQ analysis, we also compared patients having subscale impairments in either eye–hand coordination or performance scores or both (abnormal group) with patients having normal scores in both these two subscales (i.e., eye–hand coordination and performance; normal group).

### Data analysis and statistics

The methods of big data analysis were used, not due to the number of the patients (final *n* = 73) but rather due to large and complex NICU database, e.g., altogether over million SpO_2_ measurements. Time series data (measurements with associated time stamps) for oxygen-related parameters were extracted from Centricity for Critical Care information system and analyzed at the Aalto University Department of Computer Science, Espoo, Finland using PostgreSQL database engine. SpO_2_ and FiO_2_ data were extracted from data logged automatically via equipment interfaces. Data for pO_2_ and pCO_2_ analysis came from laboratory information system via Centricity for Critical Care information system interface. Supplementary oxygen time series was created by combining automatically logged FiO_2_ from ventilator interface when available (mechanical ventilation, synchronized nasal ventilation) and manually entered FiO_2_ for patients on nasal continuous positive airway pressure or high flow nasal cannulas, where no equipment interface was available. The time series data were preprocessed by removing out-of-range values caused by, e.g., missing or misplaced sensors and monitoring equipment drifting out of calibration. The data were correlated with WM injury, MEG-SII abnormalities (both at TEA), and Hempel neurological examination and GMDS quotients (at 2 years of corrected age). Student’s *T* test was used for statistical comparisons. A *p* value <0.05 was considered significant.

## Results

### Patient characteristics

The study design is shown in Fig. 1 and the clinical characteristics of the ELGANs (final *n* = 73) in Table 1A, B. WM (at TEA) was classified as abnormal in 38%, MEG-SII (at TEA) in 36%, Hempel neurological examination (at 2 years of corrected age) in 61%, and GMDS quotients in 41% of the patients. These relatively large percentages of infants in the abnormal groups are likely to be explained by the study design with classifying infants even with mild alterations in the main abnormal groups. In the subgroup analysis, there were 6 infants with severe WM changes (8.7%) and 6 with major neurologic impairment in Hempel assessments (CP, 9.1%).

In comparison with infants with normal WM, those with WM abnormalities were more often males, had tendency toward lower Apgar scores, had more often suffered from respiratory distress syndrome (RDS), and had more intraventricular hemorrhage (IVH) findings in ultrasound examinations (Table 1A). The ELGANs with severe WM changes additionally suffered more from bronchopulmonary dysplasia (BPD) (data not shown (DNS)). The patients with abnormal Hempel neurological examination were more often males (Table 1B), and the ones with major neurologic impairment (CP) also had more IVH (DNS) than the infants with normal Hempel assessment. The infants with low GMDS scores, in turn, had more retinopathy of prematurity than those with normal scores (Table 1B).

The infants with abnormal WM, MEG-SII, Hempel, or low GMDS did not significantly differ from those without these abnormalities in terms of GA, birth weight, being small for gestational age (SGA), being twins, or other parameters shown in Table 1. In addition, none of these groups statistically significantly differed from the other groups in regard to age, parity, and the smoking status of the mother; being exposed to preterm premature rupture of membranes, chorioamnionitis, pre-eclampsia, gestational diabetes, cesarean section; being extubated by day 3; or treatment for patent ductus arteriosus (DNS). The infants with abnormal WM and/or MEG-SII had tendencies toward having received more postnatal dexamethasone than the ones with normal imaging, but findings were not statistically significant (abnormal WM 19% vs normal WM 9%, *p* = 0.07 and MEG-SII 14 vs 2%, *p* = 0.09).

### Severe WM changes and abnormal MEG-SII were associated with adverse neurodevelopment

The severe WM changes at TEA were associated with abnormalities in GMDS and Hempel assessments at two years of corrected age (all *p* < 0.05, DNS). The abnormal WM group that included also minor WM changes was not significantly associated with the 2-year neurodevelopmental assessments (GMDS or Hempel, DNS).

The abnormal MEG-SII findings (at TEA) were significantly associated with worse outcomes in 2-year GMDS, which is consistent with our previous findings.^18^ Here, with a larger study group than in our previous study,^18^ also the findings with the subscales were statistically significant: the infants with abnormal MEG-SII had significantly lower GMDS SQ scores in locomotor, personal and social, hearing and language, eye–hand coordination, and performance (all *p* < 0.05, DNS). The patients with abnormal MEG-SII findings tended also to have poorer Hempel outcomes, although the finding was not statistically significant (*p* = 0.07).

### Oxygen saturations and partial pressures of oxygen during the first 3 days of life in ELGANs with later WM, MEG-SII, and neurodevelopmental abnormalities

**Fig. 2:**
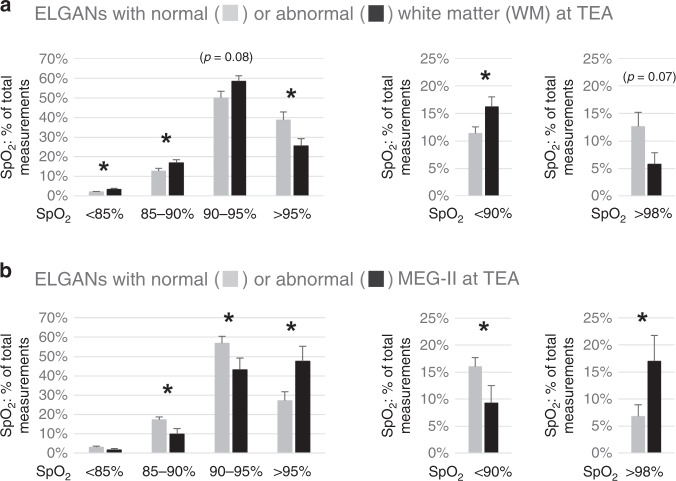
Oxygen saturations (first 3 days). ELGANs with WM changes exhibit lower and with MEG-SII abnormalities higher SpO_2_ than those with normal findings. **a** SpO_2_ (% of total measurements, mean +/− SEM, *p < 0.05) in ELGANs with normal (*n* = 43) or abnormal (*n* = 26) WM and **b** normal (*n* = 25) or abnormal (*n* = 14) MEG-SII.

During the first 3 days of life, the infants who at TEA displayed WM abnormalities showed lower average SpO_2_ (92.8% ± 0.3 vs 93.8% ± 0.3; *p* < 0.05. DNS), had more SpO_2_ <90% (*p* < 0.05) and <85% (*p* < 0.05), and less SpO_2_ >95% (*p* < 0.05) than the patients with normal WM (Fig. 2a). The cumulative times (percentage of time during the first 3 days) in patients with abnormal and normal WM having SpO_2_ <85% were 3.5 vs 2.1% (*p* < 0.05), SpO_2_ <90% 16.3 vs 12.2% (*p* < 0.05), SpO_2_ 90–95% 54.1 vs 46.0% (*p* = 0.06), SpO_2_ >95% 25.5 vs 38.3% (*p* < 0.05), and SpO_2_ >98% 5.8 vs 12.8% (*p* = 0.06), respectively. Thus, the ELGANs with abnormal WM had spent more time with lower SpO_2_ than the infants with normal WM. The subgroup with severe WM changes showed similar findings and had lower oxygen saturations, i.e., lower average SpO_2_ and more SpO_2_ measurements <90% and <85%, as well as higher cumulative times spent with SpO_2_ <90% and <85%, than the infants with normal WM at TEA (all *p* < 0.05, DNS).

The infants with abnormal MEG-SII, in turn, had higher average SpO_2_ (94.5% ± 0.4 vs 92.8% ± 0.4 *p* < 0.05, DNS) and had more SpO_2_ >95% (*p* < 0.05) and >98% (*p* < 0.05), as well as fewer SpO_2_ <90% (*p* < 0.05), than the patients with normal MEG-SII findings (Fig. 2b). They also stayed less within current oxygen saturation target ranges (90–95%, *p* < 0.05). Furthermore, their cumulative times (percentage of time during the first 3 days) when having higher SpO_2_ (>95% and >98%) were greater. The proportional times (percentage of time) in patients with abnormal and normal MEG-SII when having SpO_2_ <85% were 1.7 vs 3.1% (NS), SpO_2_ <90% 9.2 vs 17.6 (*p* < 0.05), SpO_2_ 90–95% 40.1 vs 51.6% (*p* < 0.05), SpO_2_ >95% 47.6 vs 26.5% (*p* < 0.05), and SpO_2_ >98% 17.1 vs 6.8% (*p* < 0.05), respectively.

The ELGANs with low GMDS SQ scores in eye-hand coordination or performance or both had significantly fewer low SpO_2_ (i.e., had higher SpO_2_) than their controls with better scores (DNS). The SpO_2_ levels in other abnormal GMDS subscales did not significantly differ from the ones with normal findings (DNS). The infants with major neurologic impairment in Hempel assessment (CP) had more SpO_2_ measurements <85% and higher cumulative times when having SpO_2_ <85% than the ones with normal Hempel examinations (all *p* < 0.05, DNS). The oxygen saturations in those abnormal Hempel groups that included also the minor impairments did not significantly differ from the group with normal assessments (DNS).

**Fig. 3:**
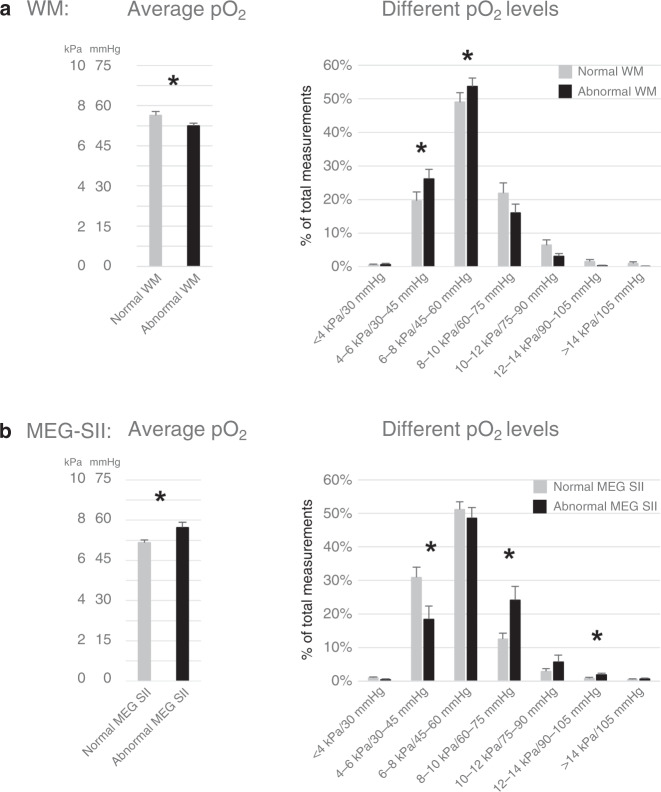
Partial pressures of oxygen (arterial pO_2_). Arterial pO_2_ (averages and percentage of total measurements in subgroups; mean +/− SEM, *p < 0.05) during the first 3 days of life in ELGANs **a** with normal (*n* = 43) or abnormal (*n* = 26) white matter (WM) and **b** with normal (*n* = 25) or abnormal (*n* = 14) MEG-SII.

Consistently with the SpO_2_ findings, the infants with WM abnormalities had lower arterial pO_2_ levels and patients with abnormal MEG-SII had higher arterial pO_2_ levels than the infants with normal WM and MEG-SII, respectively, during the first 3 days of life (Fig. 3). Also, the ELGANs with low SQ scores in locomotor, eye–hand coordination, or performance had significantly more high and fewer low pO_2_ levels (*p* < 0.05, DNS) than the ELGANs with normal scores (DNS). The arterial pO_2_ levels of the other abnormal GMDS SQs or Hempel examinations did not significantly differ from the ones with normal examinations (DNS).

### Patients with abnormal WM findings and major neurologic impairment in Hempel assessment (CP) had higher and patients with abnormal MEG-SII or low GMDS had lower need for supplemental oxygen (FiO_2_)

**Fig. 4:**
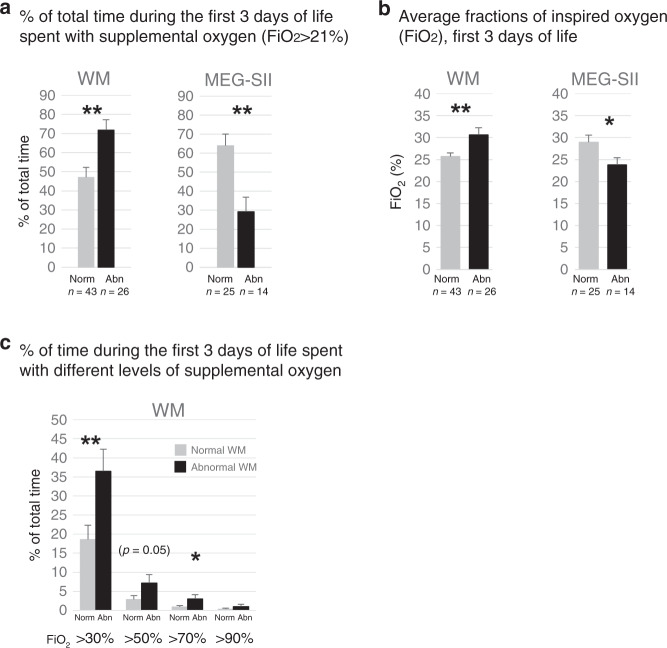
Supplemental oxygen (FiO_2_, first 3 days). ELGANs with WM injury have higher and with abnormal MEG-SII lower need for FiO_2_ than those with normal findings. **a** Time spent with FiO_2_ > 21% (%, mean +/− SEM); **b** average FiO_2_ (%, mean +/− SEM); and **c** percentage of time (%, mean +/− SEM) spent with different FiO_2_ levels. *p < 0.05, **p < 0.01.

The patients with WM abnormalities had spent more time (percentage of time) with supplemental oxygen (FiO_2_ >21%) and had required higher average FiO_2_ than the ones with normal WM (Fig. 4). The findings with severe WM abnormalities were similar (*p* < 0.01, DNS). Also the proportional times with FiO_2_ >30% (*p* < 0.01) and >70% (*p* < 0.05) were higher in infants with abnormal WM (Fig. 4c) and with severe WM outcomes (*p* < 0.01, DNS) than in those with normal WM. The ELGANs with major neurologic impairment in Hempel assessment (CP) also had a higher need for FiO_2_, i.e., they had higher proportional times with FiO_2_ >70% (*p* < 0.01) and >90% (*p* < 0.05) than the ones with normal Hempel assessments (DNS).

In turn, the infants with abnormal MEG-SII had spent less time with supplemental oxygen and received less FiO_2_ than the patients with normal MEG-SII (Fig. 4a, b). Also the group with lower GMDS scores spent significantly less time with FiO_2_ >50% than the ones with better scores (*p* < 0.01; DNS).

To investigate the role of excessive oxygen administration, we analyzed the use of supplemental oxygen when the saturations were >95% or >98%. During the first 3 days of life, the cumulative times when patients received supplemental oxygen while having SpO_2_ >95% or >98% were not statistically significantly different between any of the study groups (DNS).

### Bradycardia (HR <100 bpm) and low oxygen saturations (SpO_2_)

**Fig. 5:**
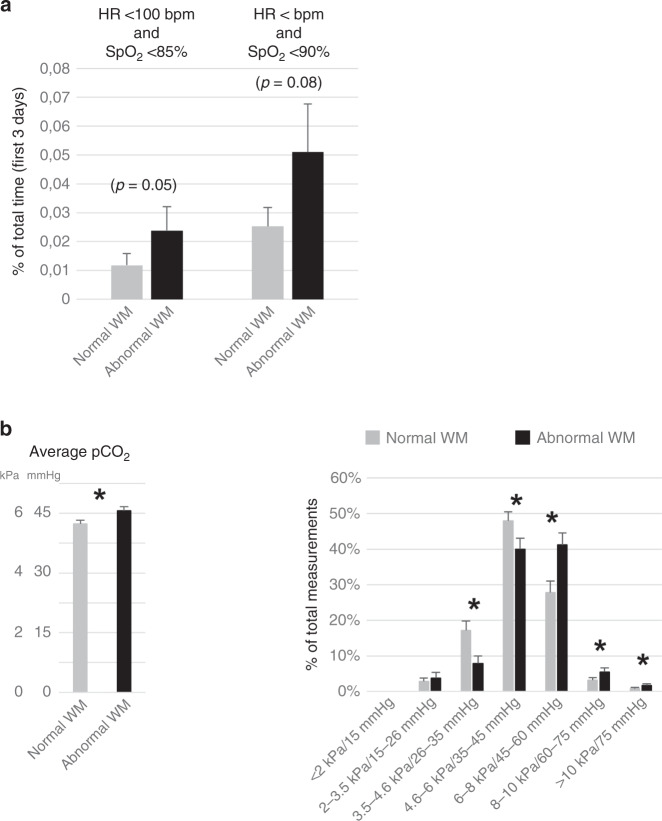
Bradycardia and low oxygen saturations (apneas), and partial pressures of carbon dioxide. **a** Bradycardia (HR <100 bpm) and low oxygen saturations (SpO_**2**_). Cumulative times, i.e., percentage of time (mean +/− SEM) during the first 3 days of life, when having at the same time heart rate (HR) below 100 bpm and SpO_2_ < 85% or SpO_2_ < 90% in ELGANs with normal (*n* = 43) or abnormal (*n* = 26) white matter (WM) in MRI at TEA. **b** Partial pressures of carbon dioxide (pCO_2_, arterial samples). Arterial pCO_2_ (averages and percentage of total measurements in subgroups; mean +/− SEM, *p < 0.05) obtained during the first 3 days of life in ELGANs with normal (*n* = 43) or abnormal (*n* = 26) white matter (WM) in MRI at TEA.

As bradycardia, in addition to oxygen-related factors, or in combination with hypoxia (apneas), is potentially associated with later neurodevelopmental impairments, we next studied the role of HR, when being <100 bpm. During the first 3 days of life, the ELGANs with WM abnormalities tended to have more bradycardia than those with normal WM. In patients with abnormal and normal WM, the proportions of HR values <100 bpm relative to all HR values (percentage of total measurements) were 0.10 and 0.05% (*p* = 0.06), and the cumulative times (percentage of time during the first 3 days) when having HR <100 min were 0.18 and 0.15% (NS), respectively. The infants with abnormal WM tended also to suffer more from the combination of bradycardia and hypoxia as measured by the cumulative time (percentage of time during the first 3 days) when having HR <100 bpm and SpO_2_ <85% (*p* = 0.05) or HR <100 bpm and SpO_2_ <90% (*n* = 0.08) than the patients with normal WM (Fig. 5a). The differences between normal and abnormal MEG-SII, Hempel, and GMDS (DQ) groups with HR data were not significant (DNS).

### Partial pressures of carbon dioxide in the study groups

In arterial blood gas analysis, the infants with WM abnormalities had higher average levels of pCO_2_ (Fig. 5b) than those with normal WM. Consistently, when analyzing different pCO_2_ levels, the patients with abnormal WM had more high level and fewer low level pCO_2_ values than those with normal WM (Fig. 5b). Regarding pCO_2_ levels, the patients with abnormal MEG-SII, low GMDS scores, or abnormal Hempel neurological examination did not statistically differ from those with normal findings (DNS).

## Discussion

Due to advancements in neonatal intensive care and increased survival of ELGANs, the prediction and prevention of later complications have become crucially important.^4–8,21–23^ In the present study, we demonstrate that the ELGANs with later WM abnormalities exhibit lower actually achieved SpO_2_ during their first 3 days of life than the infants with normal WM. In turn, the results are different with infants having abnormal MEG-SII responses, who have higher early SpO_2_ than ELGANs with normal MEG-SII. We also show that both these abnormalities, i.e., severe WM changes and abnormal MEG-SII at TEA, correlate with adverse neurodevelopment at 2 years of corrected age. The role of WM injury in unfavorable development is in accordance with previous publications.^8,17,20–23^ The SII response, in turn, is considered as an indicator of higher intracortical processing, and the absence of this response may reflect overall reduced corticocortical connectivity.^19^ The findings with WM and oxygen-linked factors are also in agreement with previous studies showing relations between low oxygen and WM injury,^1,20,21,27^ although hyperoxia has also been suggested to be harmful to WM.^28^ In terms of oxygen parameters and MEG-SII responses in infants, there are no previous data and the present findings are novel.

Consistent with the SpO_2_ findings, the achieved oxygen levels in arterial samples (pO_2_) in ELGANs with WM abnormalities were lower and with MEG-SII and GMDS abnormalities higher than in their normal controls. Thus, despite technical, limit setting, and averaging challenges with pulse oximetry,^14,29^ and discontinuous data sampling with arterial pO_2_ levels, obtaining similar results with both methods strengthens the results. The other limitations of the study include the number of patients, i.e., although the amount of data from the database was big, the number of patients (final study population of 73 patients) may not have been powerful enough for discovering all clinically significant factors. Furthermore, retrospective investigations always have limitations. In terms of updates in later devices, we consider the data obtained from the monitoring devices of our study compatible, as, e.g., the limited setting and averaging challenges still exist with the updated pulse oximetry. The possible role of compliance with pre-established SpO_2_ ranges by the stuff was not determined, but one could assume that the potential effects of compliance would be equally targeted to all different groups and thus compliance would not explain the observed differences between the patient groups.

The mechanisms underlying the opposite findings in early oxygen levels in patients with later WM injury and with abnormal MEG-SII responses are unclear. Only two of the patients had both abnormal WM and abnormal MEG-SII responses, whereas the others had either abnormal WM or MEG-SII. This suggests that the noxae causing these two conditions are likely to be different. Indeed, certain cell types and tissue structures have been shown to be more sensitive to hypoxia and others to hyperoxia. Hypoxia can, e.g., lead to proinflammatory and oxidative stress,^1^ increased production of hypoxia-inducible factors,^30^ and WM injury.^21^ Hyperoxia, in turn, has an impact on oxidative stress, changes in blood flow, disruption on neural plasticity and myelination, and may contribute to the encephalopathy of prematurity.^1,28,31^ Thus, the present results may indicate certain brain structures being more vulnerable to hypoxia and others to higher oxygen levels.

The administration of supplemental oxygen (FiO_2_) seemed, at first glance, to have been clinically appropriate, as the patients with lower SpO_2_ (abnormal WM) were supplied with higher FiO_2_ and the patients with higher SpO_2_ (abnormal MEG-SII) with less supplemental oxygen (Fig. 4). Moreover, the cumulative times of inappropriate oxygen administration did not differ between the groups. However, the administration of appropriate FiO_2_ in the NICU may not have been dynamic enough as the actually achieved oxygen saturations were relatively often out-of-the-target range (Fig. 2). Having said that, certain high SpO_2_ levels were not iatrogenically caused as some infants had high SpO_2_ at room air, i.e., without supplementary oxygen. Nevertheless, those occasions may still have been harmful, as even at room air the oxygen exposure is higher than that encountered in corresponding fetal period in utero.^2,10,32^

Other than oxygen-associated factors are undoubtedly likely to be involved in causing neurological impairments in ELGANs. In the present study, the infants with WM abnormalities had lower Apgar scores, more RDS, BPD, and IVH, and thus seem to have been in more serious general condition during the NICU period, than those with normal WM. Moreover, they were more often male, in whom the antioxidant defense maturation is shown to be delayed specifically when born preterm.^32^ All these factors may have affected the later outcomes of these ELGANs. The SGA or twin infants, in turn, were not significantly overrepresented in the abnormal groups, but the study is limited by the number of patients and may not have been powerful enough to discover all clinically significant factors.

There are several additional factors that may play roles in inducing neurodevelopmental impairments in ELGANs. Indeed, in our study, the partial pressures of carbon dioxide in patients with abnormal WM were higher than in those with normal WM. This is in accordance with previous studies showing the involvement of hypercarbia and pCO_2_ fluctuations in adverse neurological outcomes.^33–37^ Furthermore, vascularization and circulation are candidates for playing important roles. For example, vascularization of specific regions and the different regulation of vascular tone by oxygen and CO_2_ have been demonstrated with different regions of retina.^38^ Moreover, although in this study the findings with bradycardia (HR <100 bpm ± low SpO_2_) did not reach statistical significance, there were trends, and apneas (low SpO_2_ and low HR) as well as intermittent hypoxemia or hyperoxemia, systemic circulatory factors (e.g., HR, blood pressure), and hemoglobin levels may play important roles and need to be evaluated in the future. Additionally, we wish to correlate the early NICU data with the neurodevelopmental outcome at later ages, such as pre-school and school ages. Naturally, prospective investigations are needed to confirm the effects of early oxygen parameters and other related factors in later neurodevelopment in ELGANs.

## Conclusions

In conclusion, lower SpO_2_ and pO_2_ levels and higher FiO_2_ need during the first 3 days of life in ELGANs associate with later WM abnormalities and adverse neurodevelopment. The infants with abnormal MEG-SII, which is associated with later low GMDS, show the opposite findings, i.e., they have higher early SpO_2_ and pO_2_ levels, than the infants with more favorable MEG-SII and GMDS outcomes. The results indicate that of brain structures WM are more vulnerable to hypoxia, whereas others, such as cortical areas, are more sensitive to hyperoxia. Although the findings have to be confirmed in further studies, they emphasize the importance of maintaining strict saturation targets during the early postnatal period.
